# Data demonstrating the anti-oxidant role of hemopexin in the heart

**DOI:** 10.1016/j.dib.2017.05.026

**Published:** 2017-05-13

**Authors:** Giada Ingoglia, Can Martin Sag, Nikolai Rex, Lucia De Franceschi, Francesca Vinchi, James Cimino, Sara Petrillo, Stefan Wagner, Klaus Kreitmeier, Lorenzo Silengo, Fiorella Altruda, Lars S. Maier, Emilio Hirsch, Alessandra Ghigo, Emanuela Tolosano

**Affiliations:** a Dept. Molecular Biotechnology and Health Sciences, University of Torino, Torino, Italy; b Dept. Internal Medicine II, University Hospital Regensburg, Regensburg, Germany; c Dept. Medicine, Università degli Studi di Verona–Azienda Ospedaliera Universitaria Integrata Verona, Verona, Italy; d Heidelberg University Hospital / EMBL Heidelberg, Heidelberg, Germany

**Keywords:** Heme, Hemopexin, Heart, Oxidative stress

## Abstract

The data presented in this article are related to the research article entitled **Hemopexin counteracts systolic dysfunction induced by heme-driven oxidative stress** (G. Ingoglia, C. M. Sag, N. Rex, L. De Franceschi, F. Vinchi, J. Cimino, S. Petrillo, S. Wagner, K. Kreitmeier, L. Silengo, F. Altruda, L. S. Maier, E. Hirsch, A. Ghigo and E. Tolosano, 2017) [1]. Data show that heme induces reactive oxygen species (ROS) production in primary cardiomyocytes. H9c2 myoblastic cells treated with heme bound to human Hemopexin (Hx) are protected from heme accumulation and oxidative stress. Similarly, the heme-driven oxidative response is reduced in primary cardiomyocytes treated with Hx-heme compared to heme alone. Our *in vivo* data show that mouse models of hemolytic disorders, β-thalassemic mice and phenylhydrazine-treated mice, have low serum Hx associated to enhanced expression of heme- and oxidative stress responsive genes in the heart. Hx^-/-^ mice do not show signs of heart fibrosis or overt inflammation. For interpretation and discussion of these data, refer to the research article referenced above.

**Specifications Table**

**Table:** 

Subject area	Health sciences
More specific subject area	Heme/iron biology
Type of data	Text file, Figures
How data was acquired	Olympus BH-2 microscope (Olympus Italia, Milan, Italy), 7300 Real Time PCR System (Applied Biosystems, Life Technologies Italia), spectrofluorimeter (Glomax, Promega Italia)
Data format	Raw, analyzed
Experimental factors	H9c2 (ATCC CRL-1446™) myoblast cell line; mouse neonatal primary cardiomyocytes; Hx^-/-^ mice; β-thalassemia mice; C57BL/6 wild-type mice
Experimental features	Gene expression was analyzed by qRT-PCR and Western blotting. Tissue inflammation was analyzed by histology and immunohistochemistry. Heme content and ROS accumulation were quantified by fluorometric methods.
Data source location	Dept. Molecular Biotechnology and Health Sciences, Torino, Italy
Data accessibility	The data are available with this article.

**Value of the data**

• These data show that the plasma protein hemopexin (Hx) limits heme accumulation within cardiac cells both *in vitro* and *in vivo*
• In mice, heme-driven oxidative stress associated to Hx exhaustion can be recovered by the administration of the anti-oxidant α-tocopherol
• These finding might be exploited in the future for the development of Hx-based drugs able to prevent cardiac heme accumulation and oxidative stress in hemolytic disorders and/or in pathologic conditions associated with heme overload

## 1 Data

**Fig. 1:**
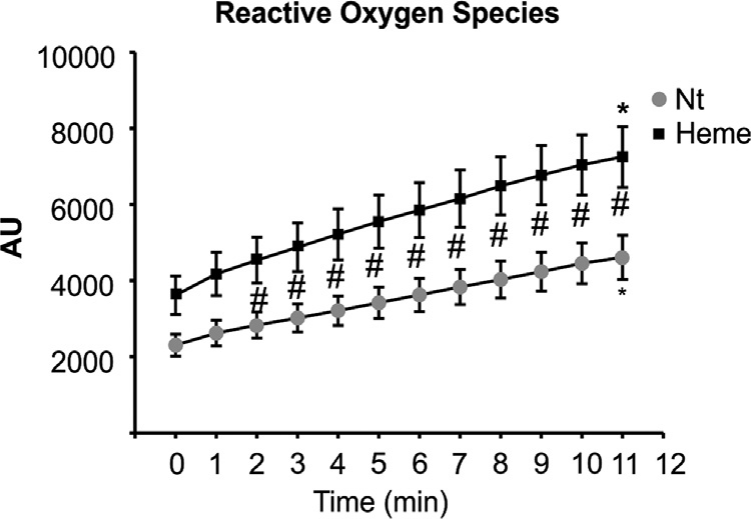
Heme promotes ROS formation in isolated adult rat cardiomyocytes. Data on isolated adult rat cardiomyocytes exposed to heme (5 µM) or vehicle (not-treated, Nt) are shown. ROS were measured by using the fluorescent dye CM-H2DCFDA (Nt, n = 22; heme, n = 17). Two-way ANOVA with Bonferroni post-test analysis was performed. *P < 0.05; #P < 0.05 (#, difference between Nt and heme-treated cells; *, difference between time 0 and time 11 in Nt and heme-treated cells).

**Fig. 2:**
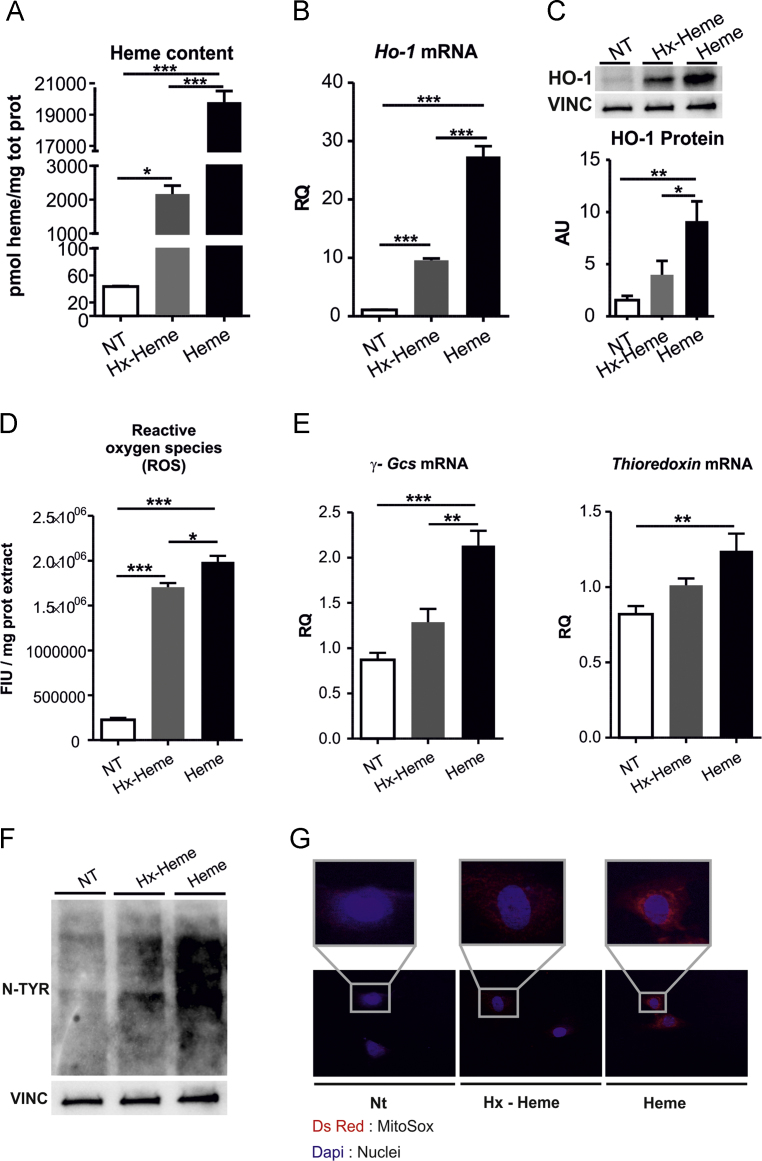
Hemopexin protects H9c2 cells from heme accumulation and ROS production. Data on H9c2 myoblasts cell line untreated (NT) or treated with either 10 µM Hx-heme complex or 10 µM heme for 8 hours, are shown. (A) Heme content. (B) qRT-PCR analysis of Ho-1 mRNA levels. (C) Western blot analysis of HO-1. (D) ROS content and (E) qRT-PCR analysis of γ-Glutamylcysteine synthetase (γ-Gcs) and Thioredoxin mRNA levels. (F) Western blot analysis of N-Tyr. (G) Immunofluorescence analysis of super-oxide radical formation (super-oxide radical was stained with Mito-sox fluorescent probe. Nuclei were stained with DAPI). Results shown are representative of three independent experiments. One-way analysis of variance with Bonferroni post-test analysis was performed. *P < 0.05; **P < 0.01; ***P < 0.001. Values represent mean ± SEM. AU, arbitrary units; RQ, relative quantity; FIU, fluorescence intensity unit.

**Fig. 3:**
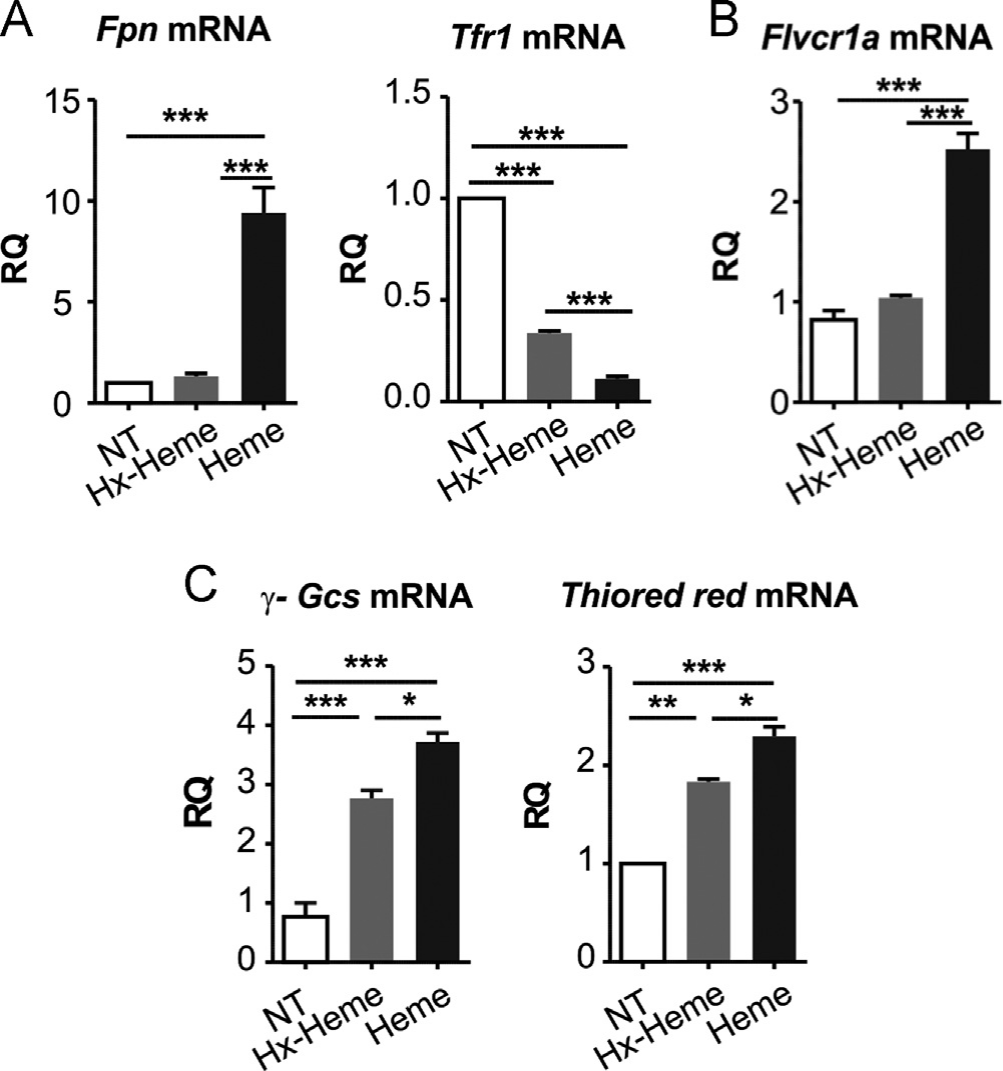
Hemopexin protects neonatal cardiomyocytes and H9c2 cells from heme accumulation and ROS formation. Data on neonatal cardiomyocytes and H9c2 cells untreated (NT) or treated with either 10 µM Hx-heme complex or 10 µM heme for 8 hours, are shown. (A, C) qRT-PCR analysis of Fpn, Tfr1, γ-Gcs and Thioredoxin reductase mRNA levels of neonatal cardiomyocytes. (B) qRT-PCR analysis of Flvcr1a mRNA levels of H9c2 cells. One-way analysis of variance with Bonferroni post-test analysis was performed. *P < 0.05; **P < 0.01; ***P < 0.001.

**Fig. 4:**
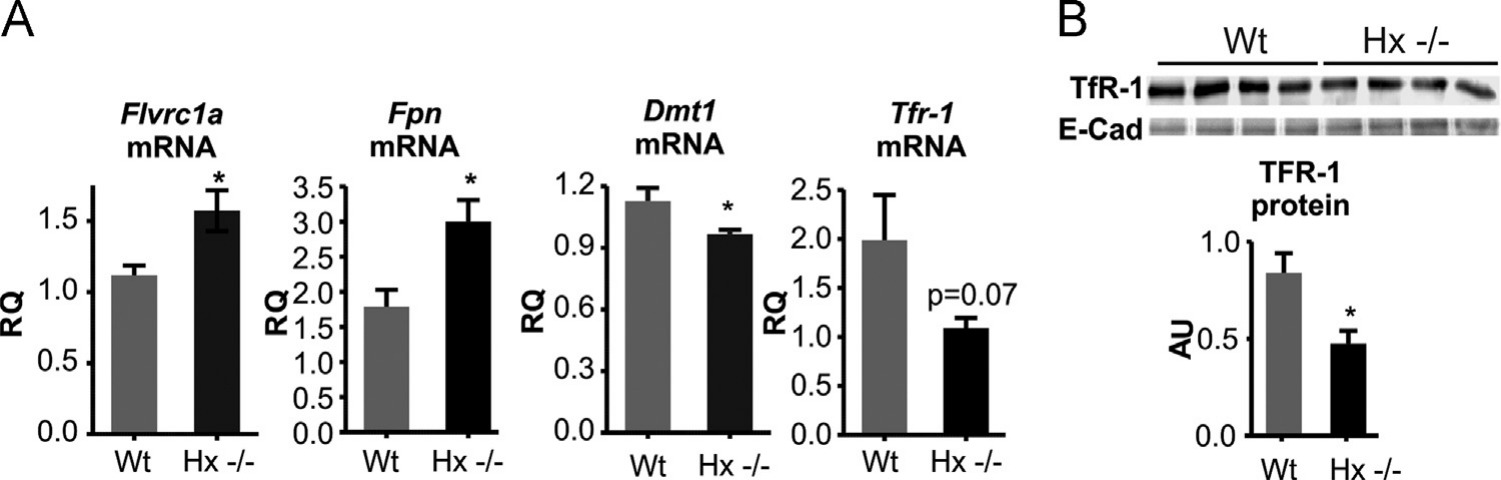
Hemopexin preserves heme homeostasis in the heart. Data on the heart of wild-type (Wt) and Hx^-/-^ mice are shown. (A) qRT-PCR analysis of Flvcr1a, Fpn, Dmt1 and Tfr1 mRNA levels. (B) Western blot analysis of Tfr1 protein. Results shown are representative of 3 independent experiments. In B, each lane represents an individual animal; E-cadherin (E-Cad) was used as loading control. Unpaired t-test analysis with Welch׳s correction was performed. Values represent mean ± SEM. *P<0.05.

**Fig. 5:**
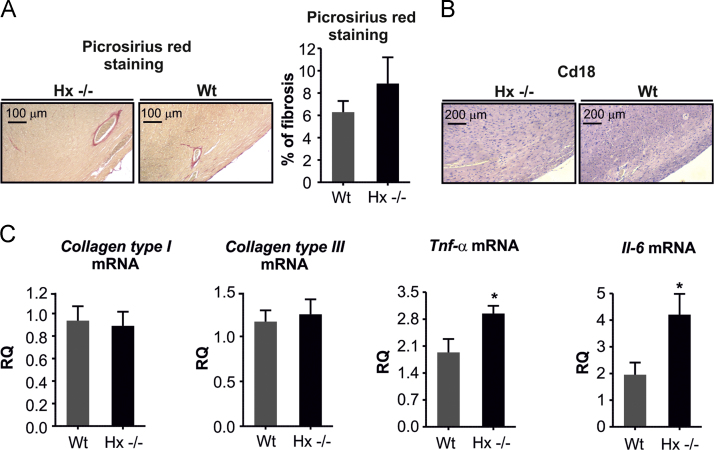
Hemopexin loss is not associated with heart fibrosis. Data on Wt and Hx^-/-^ mice are shown. (A) Representative Picrosirius Red staining of heart sections from a Wt and an Hx^-/-^ mouse. ImageJ analysis of Picrosirius Red stained sections is shown on the right. (B) Immunohistochemistry analysis of CD18 expression on heart sections of a Wt and a Hx-/- mouse. (C) qRT-PCR analysis of collagen type I and III, Tnf-α and IL6 mRNA levels in the heart (n = 5). Unpaired t-test analysis with Welch׳s correction was performed. Values represent mean ± SEM. *P<0.05.

Data show that heme induced ROS production in primary cardiomyocytes (Fig. 1). Hx limited heme accumulation within H9c2 cell (myoblast cell line) and prevented ROS production. H9c2 cells were treated with heme alone or heme bound to Hx,and heme content, ROS production, the expression of heme- and oxidative stress responsive genes and markers of oxidative stress were evaluated (Fig. 2). These data were confirmed in primary cardiomyocytes isolated from neonatal mice and treated with either heme alone or heme-Hx (Fig. 3) and, indirectly in the heart of Hx^-/-^ mice (Fig. 4). Data in Fig. 5 show that the heart of Hx^-/-^ mice, despite of heme accumulation and elevated ROS [1], did not show sign of fibrosis and inflammation apart a slight increase in the level of Tumor Necrosis Factor (TNF)α and Interleukin (IL)-6 mRNAs.

**Fig. 6:**
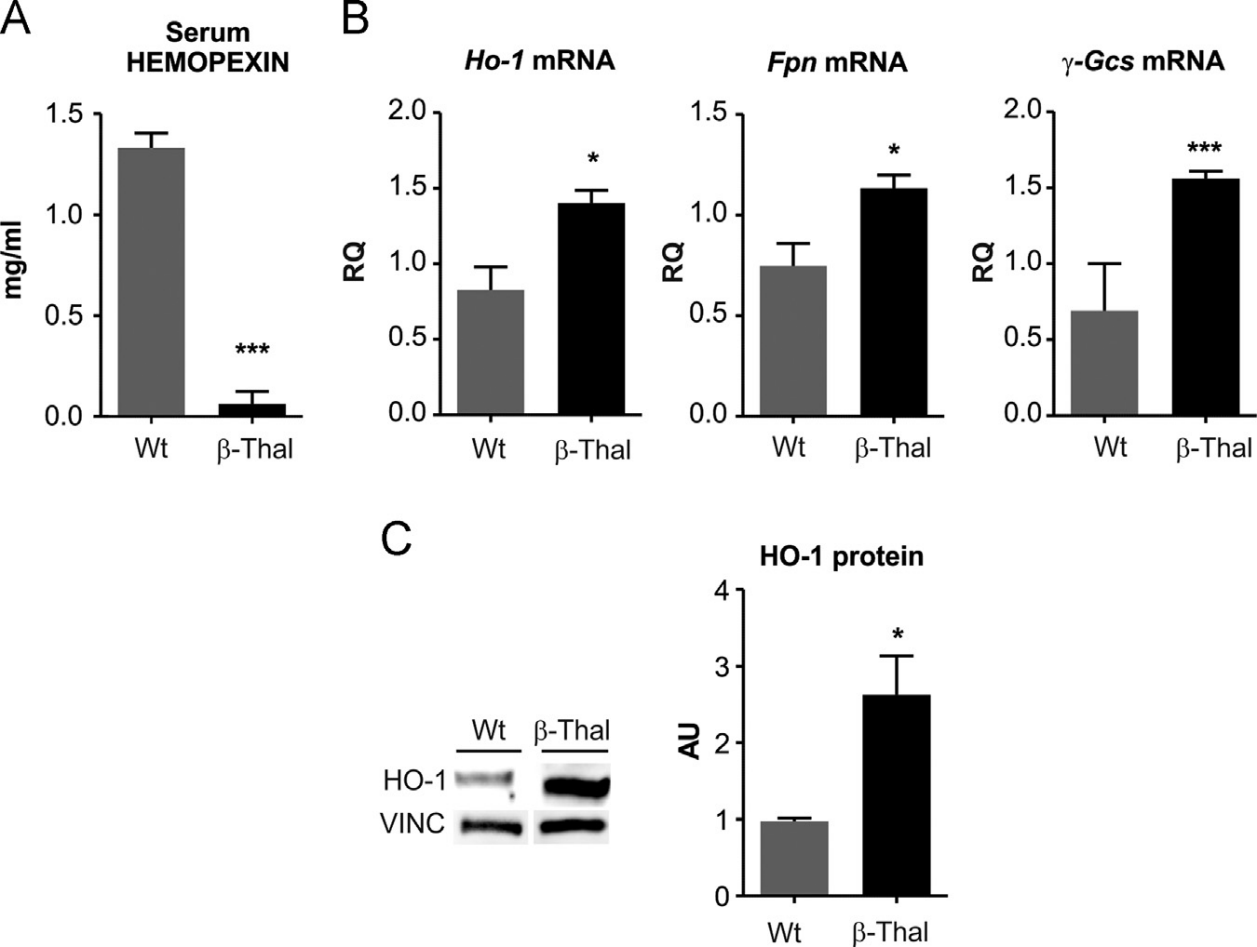
β-thalassemic mice are hemolytic and accumulate heme in the heart. Data on Wt and β-thalassemic (β-Thal) mice are shown. (A) ELISA quantification of serum Hx. (B) qRT-PCR analysis of Ho-1, Fpn and γ-Gcs mRNA levels in the heart. (C) HO-1 western blot analysis. Unpaired t-test analysis with Welch׳s correction was performed. ^⁎^P < 0.05; ^⁎⁎⁎^P < 0.001. Values represent mean ± SEM.

**Fig. 7:**
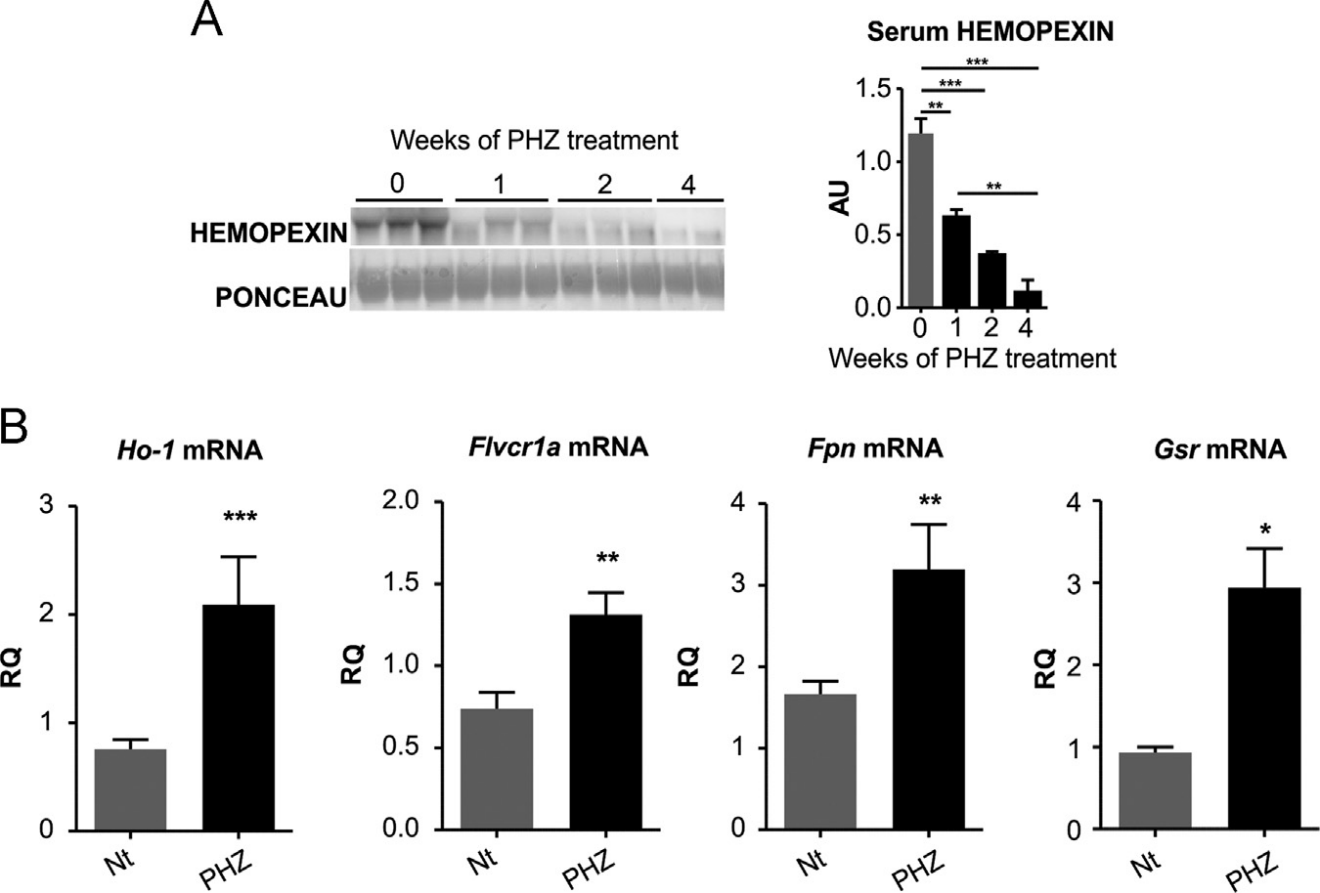
PHZ-treated mice show an alteration of heme- and oxidative stress-responsive genes in the heart. (A) Western blot of serum Hx of untreated (0) or PHZ-treated Wt mice at 1, 2 or 4 weeks of treatment. (B) qRT-PCR analysis of Ho-1, Flvcr1a, Fpn, Gsr, mRNA levels in the heart of untreated or PHZ-injected mice after 4 weeks of treatment. In A, one-way ANOVA with Bonferroni post-test analysis was performed; in B, unpaired t-test analysis with Welch׳s correction was performed. *P < 0.05; ** P < 0.01; ***P < 0.001.

**Fig. 8:**
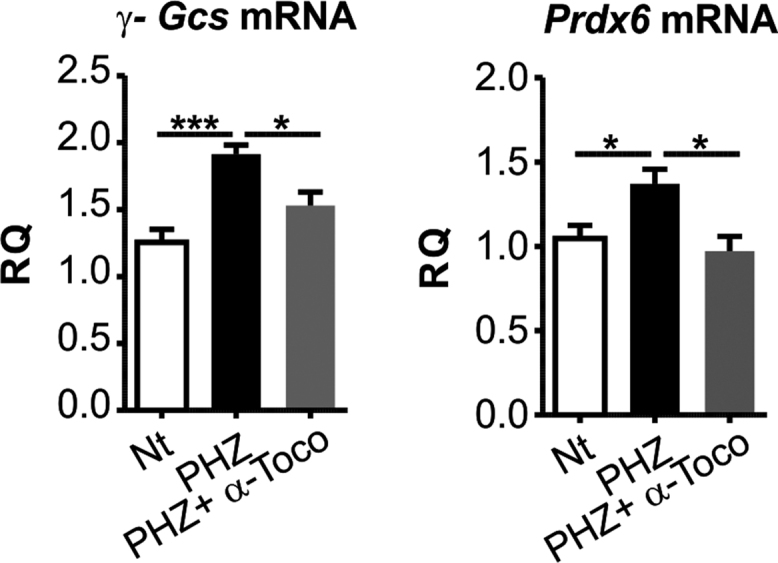
α-tocopherol protects the heart against PHZ-mediated oxidative stress. Data on the heart of PHZ-treated Wt mice administered or not with α-tocopherol are shown. (A) qRT-PCR analysis of γ-Gcs and peroxiredoxin 6 (Prdx6) mRNA levels of PHZ- and PHZ-α-tocopherol-treated mice (n=7) 4 weeks after the treatment. One-way analysis of variance with Bonferroni post-test analysis were performed. ^⁎^P < 0.05; ^⁎⁎⁎^P < 0.001. Values represent mean ± SEM. RQ, relative quantity.

*In* *vivo*, Hx depletion in mouse models of hemolytic disorders, β-thalassemic mice and phenylhydrazine (PHZ)-treated mice, was associated with heme accumulation and oxidative stress in the heart. Data show that in β-thalassemic mice, low Hx serum level, was associated to increased expression of heme- and oxidative stress responsive genes in the heart (Fig. 6). The same occurred in PHZ-treated mice (Fig. 7). Administration of the anti-oxidant α-tocopherol to PHZ-treated mice normalized the expression of anti-oxidant genes (Fig. 8).

## 2 Experimental Design, Materials and Methods

### 2.1 Cells and treatments

H9c2 (ATCC CRL-1446™) cells and primary cardiomyocytes, isolated from neonatal mice were treated with either 10 µM Hx-heme complex or 10 µM heme for 8 hours. Primary adult rat cardiomyocytes were treated with 5 µM heme or vehicle for 15 min. Heme and Hx-heme complex were prepared as described [1].

### 2.2 Mice and treatments

Hx^-/-^ mice and β-thalassemia mice were previously generated [2], [3], [4], [5], [6], [7]. C57BL/6 wild-type mice were administered intraperitoneally (i.p.) with 25 mg/kg phenylhydrazine (PHZ, Sigma-Aldrich, Saint Luis, USA) twice a week for 4 weeks. PHZ-treated mice were injected i.p. with 400 mg/kg α-tocopherol (Sigma-Aldrich) dissolved in corn oil or with vehicle on the day of PHZ injection.

### 2.3 Gene expression analysis

Total RNA, from cells or tissues, was extracted using Pure Link RNA Mini Kit (Ambion, Life Technologies Italia, Milano, Italy). qRT-PCR was performed on a 7300 Real Time PCR System (Applied Biosystems, Life Technologies Italia). Primers and probes were designed using the ProbeFinder software (http://www.roche-applied-science.com).

For Western blotting, tissue and cell proteins were extracted as reported [1]. Fifty µg total protein or 0.25 µL mouse serum were separated on SDS-PAGE and immunoblotted using antibodies against HO-1 (dilution 1:300, Enzo Life Sciences), Hx (1:1000) [8], N-Tyr (1:1000, Merck Millipore).

### 2.4 Heme content and ROS accumulation

Heme content in cells and tissues was quantified fluorometrically by the method of Sassa [9], [10]. Accumulation of ROS in heart homogenates or cells was assessed by using either 29,79-dichlorodihydrofluoroscein diacetate (H2DCFDA; Molecular Probes, Inc., Eugene, OR) [11] or MitoSOX (ThermoFisher Scientific, Waltham, MA USA).

### 2.5 Immunohistochemistry and histology

Hearts were processed as described and analyzed by immunohistochemistry with an anti- CD18 antibody (1:100, Biolegend). For collagen quantification, tissue sections were stained with Picrosirius Red and analyzed by Image J program.

### 2.6 Statistical Analysis

Results were expressed as mean ± SEM. Comparisons between 2 groups were performed with 2-sided Welch t tests and among >2 groups with 1- or 2-way ANOVA followed by the Bonferroni post-test (GraphPad software Inc, La Jolla, CA). A value of P<0.05 was considered significant.
