# Supplementary material for: Data demonstrating the anti-oxidant role of hemopexin in the heart
Source: Data Brief. 2017 May 13;13:69–76. doi: 10.1016/j.dib.2017.05.026 (PMC5443894; doi:10.1016/j.dib.2017.05.026)
Supplement: Supplementary file 1 — Supplementary material [file mmc1.docx]

**Author Disclosure Statement**

ET has received research funding from CSL Behring. All the other authors have nothing to declare.
